# Long-term temporal trends and seasonal variations in Bell’s Palsy and severity: an STL time-series approach

**DOI:** 10.1016/j.bjorl.2026.101892

**Published:** 2026-09-02

**Authors:** Vural Akın, Hasan Yasan, Gökhan Keskin, Furkan Yeşilkuş, Mehmet Emre Sivrice, Yusuf Çağdaş Kumbul

**Affiliations:** Süleyman Demirel University Faculty of Medicine, Department of Otorhinolaryngology-Head and Neck Surgery, Isparta, Turkey

**Keywords:** Bell palsy, Facial paralysis, Seasons, Seasonal variation

## Abstract

**Objective:**

Bell's palsy is the most common form of acute peripheral facial palsy; however, its etiology remains unclear. Environmental and seasonal factors have been suggested as potential triggers, but previous studies have reported conflicting results. The aim of this study was to evaluate the long-term temporal and seasonal patterns of Bell's palsy using a 10-year dataset and seasonal-trend decomposition using locally estimated scatterplot smoothing time-series analysis.

**Methods:**

This retrospective study analyzed data of patients with Bell's palsy who presented between 2015 and 2024. Bell's palsy severity according to the House-Brackmann scale and presentation dates were assessed by month, season, and year. Chi-Square tests and seasonal-trend decomposition using locally estimated scatterplot smoothing time-series analysis were used to assess temporal and seasonal patterns.

**Results:**

A total of 352 patients were included in the study (53.9% male; mean age, 46.5 ± 18.3 years). The highest frequency of presentations occurred in October, whereas the lowest occurred in February, with no significant monthly or seasonal differences (p > 0.05). Seasonal-trend decomposition suggested small recurring fluctuations, with a modest increase in March compared with February, and persistence through April and May. Paralysis severity showed no temporal variation.

**Conclusion:**

In this single-center clinic-based study, Bell’s palsy presentations showed no statistically significant monthly, seasonal, or annual variation, and severity was not associated with temporal categories. Seasonal-trend decomposition using locally estimated scatterplot smoothing time-series analysis suggested only minor recurrent fluctuations. Because direct meteorological and environmental exposure data were not analyzed, these findings should be interpreted as descriptive temporal presentation patterns rather than evidence supporting or refuting specific environmental triggers.

## Highlights

• Bell’s palsy presentations were evaluated over a 10-year period in a tertiary clinic.
• No statistically significant monthly, seasonal, or annual variation was observed.
• House-Brackmann severity did not differ according to temporal categories.
• STL decomposition suggested minor recurrent fluctuations, interpreted as exploratory.
• The study describes clinic-based presentation patterns, not environmental triggers.

## Introduction

The most common clinical presentation of peripheral facial paralysis, Bell’s Palsy (BP), is defined as an acute-onset idiopathic paralysis of the facial nerve. The annual incidence of BP has been reported to range between 11.5 and 53.3 per 100,000 individuals, and its etiopathogenesis has been associated with anatomical variations, viral infections, ischemia, inflammation, and exposure to cold.^1^^,^^2^ The severity of BP has prognostic significance, as complete paralysis is associated with a lower response rate to treatment. Reported treatment response rates among patients with BP range from 61% to 94%. In addition to the psychological distress caused by facial disfigurement, secondary complications such as corneal damage and synkinesis may also occur as a consequence of BP. Although the optimal treatment remains debatable, corticosteroids, antiviral agents, physiotherapy, and decompression surgery are commonly used in clinical practice.^1^ Recent reviews continue to emphasize the multifactorial and largely idiopathic nature of BP, with early corticosteroid therapy remaining the cornerstone of medical treatment.^1^, ^2^, ^3^, ^4^ Despite advances in diagnostic and therapeutic approaches, the relative contributions of viral, inflammatory, ischemic, anatomical, and environmental mechanisms remain incompletely understood.^1^^,^^3^^,^^4^

The relationship between BP and meteorological variables has been examined in numerous studies. The potential roles of factors such as air temperature, atmospheric pressure, humidity, and wind speed have been investigated. Additionally, the monthly and seasonal distribution of BP cases throughout the year has been evaluated in studies conducted across different countries. Although many reports have indicated an increased incidence of BP during the colder winter months, when air temperatures are lower, the findings vary considerably, even within the same country.^1^^,^^5^, ^6^, ^7^, ^8^, ^9^, ^10^, ^11^, ^12^, ^13^, ^14^ However, only a limited number of studies have specifically evaluated BP severity in relation to these meteorological parameters.^7^^,^^15^^,^^16^

In this study, we aimed to retrospectively analyze the distribution and severity of BP cases according to month, season, and year among patients who presented to a tertiary otorhinolaryngology clinic between January 1, 2015, and December 31, 2024. A more detailed examination was conducted using seasonal-trend decomposition using locally estimated Scatterplot Smoothing (STL) time-series analysis to assess seasonal variations. This approach was used to provide an exploratory and descriptive assessment of long-term temporal patterns and potential within-year fluctuations in clinic-based BP presentations.

## Methods

This retrospective study was conducted in the Department of Otorhinolaryngology, Faculty of Medicine, Süleyman Demirel University, after receiving approval from the Ethics Subcommittee of the Health Sciences Ethics Committee of Süleyman Demirel University Rectorate (Approval nº50, Date: July 4, 2025). The identities of all the patients were kept confidential, and the data were anonymized before analysis. The study was conducted in accordance with the principles of the Declaration of Helsinki. Within the scope of the study, data from patients who presented with peripheral facial paralysis to the Otorhinolaryngology Clinic between January 1, 2015, and December 31, 2024, were analyzed. These data were obtained from the hospital’s digital archive system.

This study was designed as a clinic-based retrospective analysis rather than a population-based incidence study. The study center is a tertiary otorhinolaryngology clinic that receives patients through direct outpatient admission and referrals from emergency departments, neurology clinics, and primary or secondary healthcare facilities. In our clinical practice, patients presenting with acute peripheral facial paralysis, either directly or by referral, are evaluated on the same day without requiring a scheduled outpatient appointment. Therefore, institutional appointment-related delays are unlikely to have substantially affected the timing of otorhinolaryngologic evaluation after presentation to our institution. Accordingly, the analyzed sample represents adult patients with BP who presented to or were referred to this tertiary otorhinolaryngology clinic during the study period. Because the study did not include a predefined population denominator or all healthcare facilities within a clearly defined catchment area, the findings should be interpreted as clinic-based presentation patterns rather than true regional incidence rates.

**Fig. 1:**
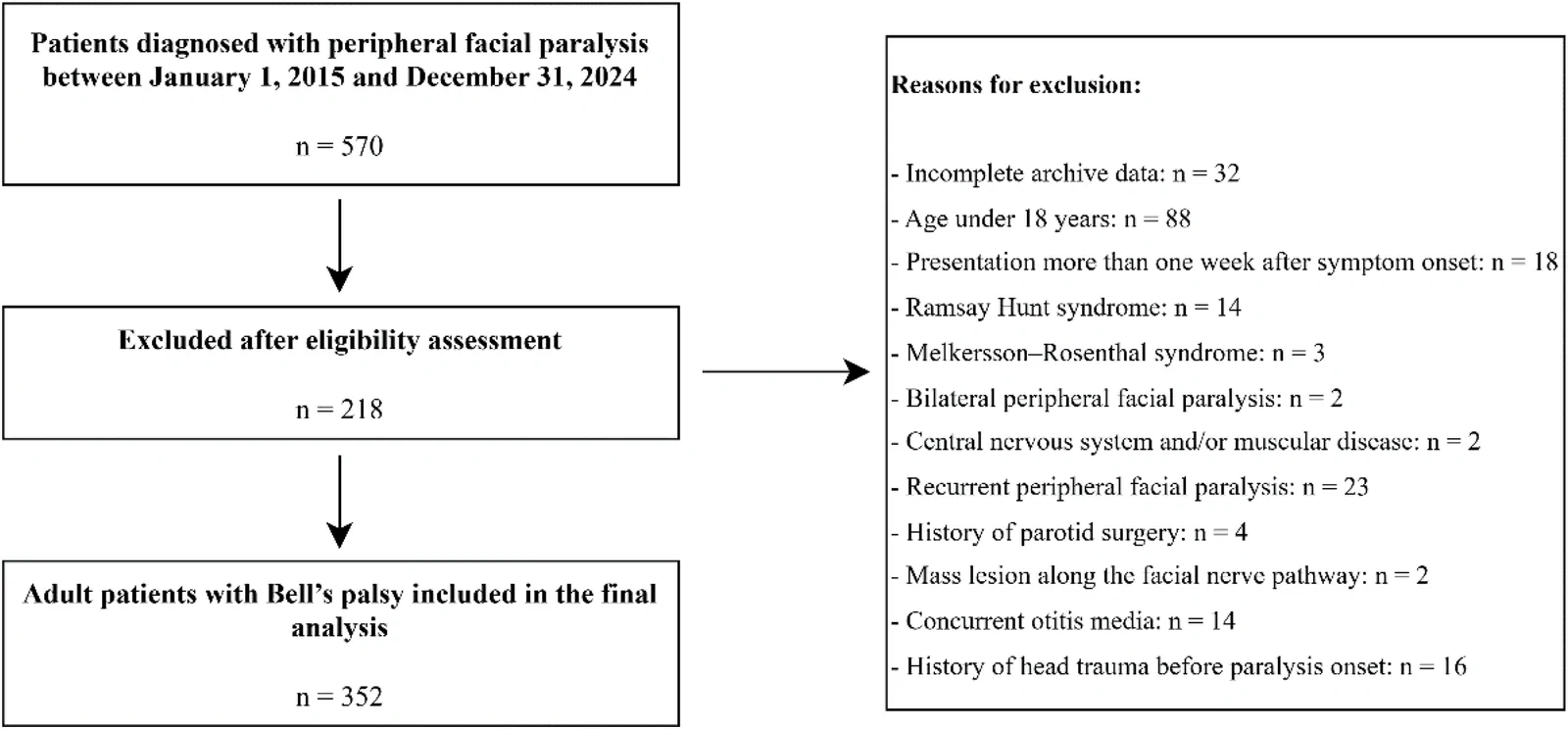
STROBE flow diagram of the patient selection process. Exclusion categories were based on the primary reason for exclusion to avoid double counting of overlapping clinical findings.

The inclusion criteria were defined as follows: complete availability of the relevant data during archive review, age >18-years, absence of physical or radiological findings that could explain the development of peripheral facial paralysis, and presentation within 1-week after the onset of facial paralysis. The exclusion criteria were: a history of head trauma before the onset of facial paralysis, facial paralysis secondary to Ramsay Hunt syndrome, accompanying hearing loss, facial paralysis due to Melkersson-Rosenthal syndrome, bilateral facial paralysis, a history of central neurological or muscular disease, recurrent peripheral facial paralysis, a history of parotid surgery, the presence of a mass lesion along the facial nerve pathway, and concurrent otitis media. The patient selection process, including the number of initially screened patients, exclusions, reasons for exclusion, and the final analytic sample, is summarized in a STROBE flow diagram (Fig. 1).

A list of patients with diagnostic codes related to peripheral facial paralysis was obtained from the hospital’s Information Technologies Department. The following data were analyzed: date of presentation, age, sex, medical history, discharge summary details, and radiological, audiological, and physical examination findings related to the respective presentation. Presentation dates were initially recorded in the day/month/year format and subsequently categorized by month, season, and year. The severity of peripheral facial paralysis was graded according to the House-Brackmann Facial Paralysis Grading System. In our country, the seasonal classification is defined as follows: spring (March, April, May), summer (June, July, August), autumn (September, October, November), and winter (December, January, February). Seasonal grouping in this study was based on this classification. Statistical analyses were performed using the obtained data, including age, sex, side of facial paralysis, House-Brackmann score, and date of presentation.

### Statistical analysis

Conventional statistical analyses were performed using IBM SPSS Statistics for Windows, version 30.0 (IBM Corp., Armonk, NY, USA). A p-value of <0.05 was considered statistically significant. The distribution of BP cases across months, seasons, and years was evaluated using the Chi-Square goodness-of-fit test to determine whether the observed frequencies differed from the expected uniform distribution. For STL analysis, individual presentation dates were aggregated into monthly case counts, generating a monthly time series from January 2015 to December 2024. The seasonal period was defined as 12-months to represent the annual cycle. STL analysis was selected because it enables the observed time series to be separated into trend, seasonal, and residual components without requiring a fixed parametric form for temporal variation.^17^ Given the relatively small number of cases within monthly intervals, the STL output was interpreted descriptively and considered exploratory rather than confirmatory evidence of seasonality. The relationship between BP severity and temporal factors was examined using the Chi-Square test of independence, applied separately to month × grade and season × grade contingency tables. Expected cell frequencies were checked to confirm test validity. Results were summarized in descriptive tables and visualized using heatmaps generated in Python with Google Colab to illustrate temporal and categorical patterns of BP case distribution and severity.

## Results

Patient selection is summarized in Fig. 1. A total of 570 patients diagnosed with peripheral facial paralysis during the study period were screened. Of these, 218 were excluded because they did not meet the eligibility criteria, most commonly because of age <18-years (n = 88), incomplete archive data (n = 32), recurrent peripheral facial paralysis (n = 23), presentation more than 1-week after symptom onset (n = 18), a history of head trauma before paralysis onset (n = 16), Ramsay Hunt syndrome (n = 14), or concurrent otitis media (n = 14). Ultimately, 352 adult patients with BP were included in the final analysis. Exclusion categories were recorded according to the primary reason for exclusion to avoid double counting of overlapping clinical findings. Among the included patients, 190 (53.98%) were male and 162 (46.02%) were female. The mean age was 46.51 ± 18.26 years (range, 18–91 years). BP affected the left side in 178 (50.57%) patients and the right side in 174 (49.43%). According to the House-Brackmann grading system, 77 (21.9%) patients had grade 2, 116 (33.0%) had grade 3, 79 (22.4%) had grade 4, 45 (12.8%) had grade 5, and 35 (9.9%) had grade 6 paralysis. Because the analyses were performed only in patients diagnosed with BP, House-Brackmann grade 1, which represents normal facial function, was not included in the tables or figures.

In the month-based analysis, the highest number of presentations was observed in October (n = 36, 10.2%), whereas the lowest number of presentations occurred in February (n = 21, 6.0%). However, the chi-square goodness-of-fit test revealed no statistically significant difference in the number of presentations across the months (p = 0.582). Therefore, the distribution of BP presentations by month was considered homogeneous (Table 1).

**Table 1:** Distribution of Bell’s palsy cases by month (2015–2024).

Month	Observed (n)	Expected (n)	Residual
January	29	29.3	−0.3
February	21	29.3	−8.3
March	35	29.3	5.7
April	34	29.3	4.7
May	34	29.3	4.7
June	31	29.3	1.7
July	27	29.3	−2.3
August	22	29.3	−7.3
September	26	29.3	−3.3
October	36	29.3	6.7
November	26	29.3	−3.3
December	31	29.3	1.7
Total	352		

Chi-Square goodness-of-fit test: p = 0.582.

In the analysis of presentations by season, the highest number of presentations was observed in spring (n = 103, 29.3%), whereas the lowest number was recorded in summer (n = 80, 22.7%). However, no statistically significant difference was identified among seasons (p = 0.279; Table 2).

**Table 2:** Distribution of Bell’s palsy cases by season (2015–2024).

Season	Observed (n)	Expected (n)	Residual
Winter	81	88.0	−7.0
Spring	103	88.0	15.0
Summer	80	88.0	−8.0
Autumn	88	88.0	0.0
Total	352		

Chi-Square goodness-of-fit test: p = 0.279.

In the year-based comparison, the number of presentations was higher than expected in 2019 and 2021, whereas it was lower than expected in 2015 and 2024. However, these differences were not statistically significant (p = 0.062). Overall, the distribution of BP cases by year demonstrated a homogeneous pattern (Table 3). The study period covered the full interval from January 1, 2015, to December 31, 2024.

**Table 3:** Distribution of Bell’s palsy presentations by year.

Year	Observed (n)	Expected (n)	Residual
2015	22	35.2	−13.2
2016	39	35.2	3.8
2017	33	35.2	−2.2
2018	41	35.2	5.8
2019	46	35.2	10.8
2020	33	35.2	−2.2
2021	46	35.2	10.8
2022	36	35.2	0.8
2023	29	35.2	−6.2
2024	27	35.2	−8.2
Total	352		

Chi-Square goodness-of-fit test: p = 0.062.

**Fig. 2:**
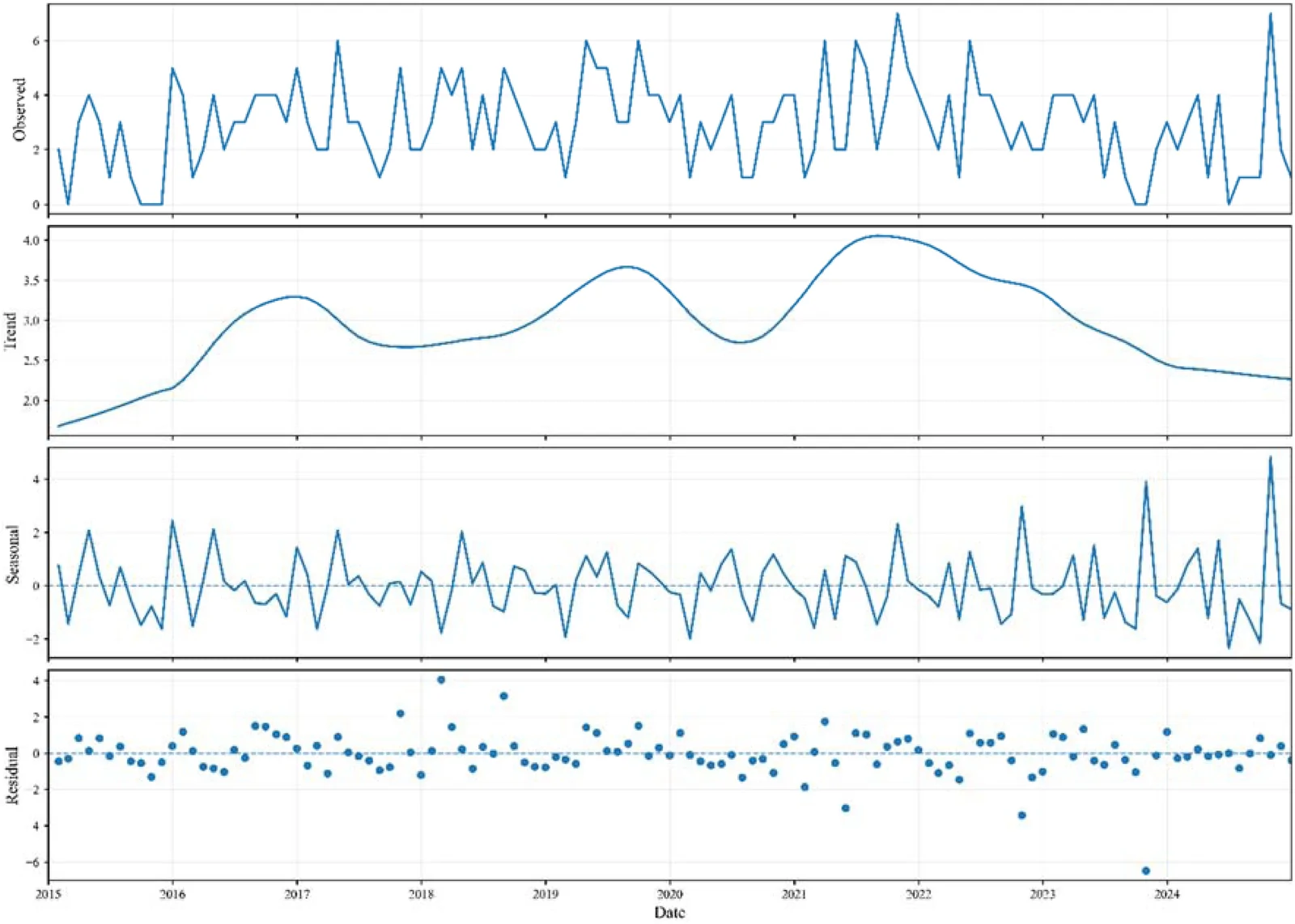
Seasonal-trend decomposition using locally estimated scatterplot smoothing graphs illustrating Bell’s palsy presentation dates.

The STL graph based on monthly case counts is presented in Fig. 2. In the STL analysis applied to the time series, monthly case counts were decomposed into four components: observed, trend, seasonal, and residual. In the overall pattern of the time series, the number of BP presentations did not show a distinct regularity over the years but rather moderate fluctuations without abrupt spikes or sharp declines. The trend component indicated a gradual increase in BP presentations starting from 2015, peaking around 2019–2020, followed by a slight decline beginning in 2021. STL analysis results suggested small-amplitude recurrent fluctuations in the seasonal component, with a modest increase that began in March compared with February and remained positive through April and May. However, these fluctuations were minor and should be interpreted cautiously because the conventional monthly and seasonal comparisons were not statistically significant and the number of cases within each monthly interval was limited. The residual component was symmetrically distributed around zero and showed no discernible temporal pattern.

**Fig. 3:**
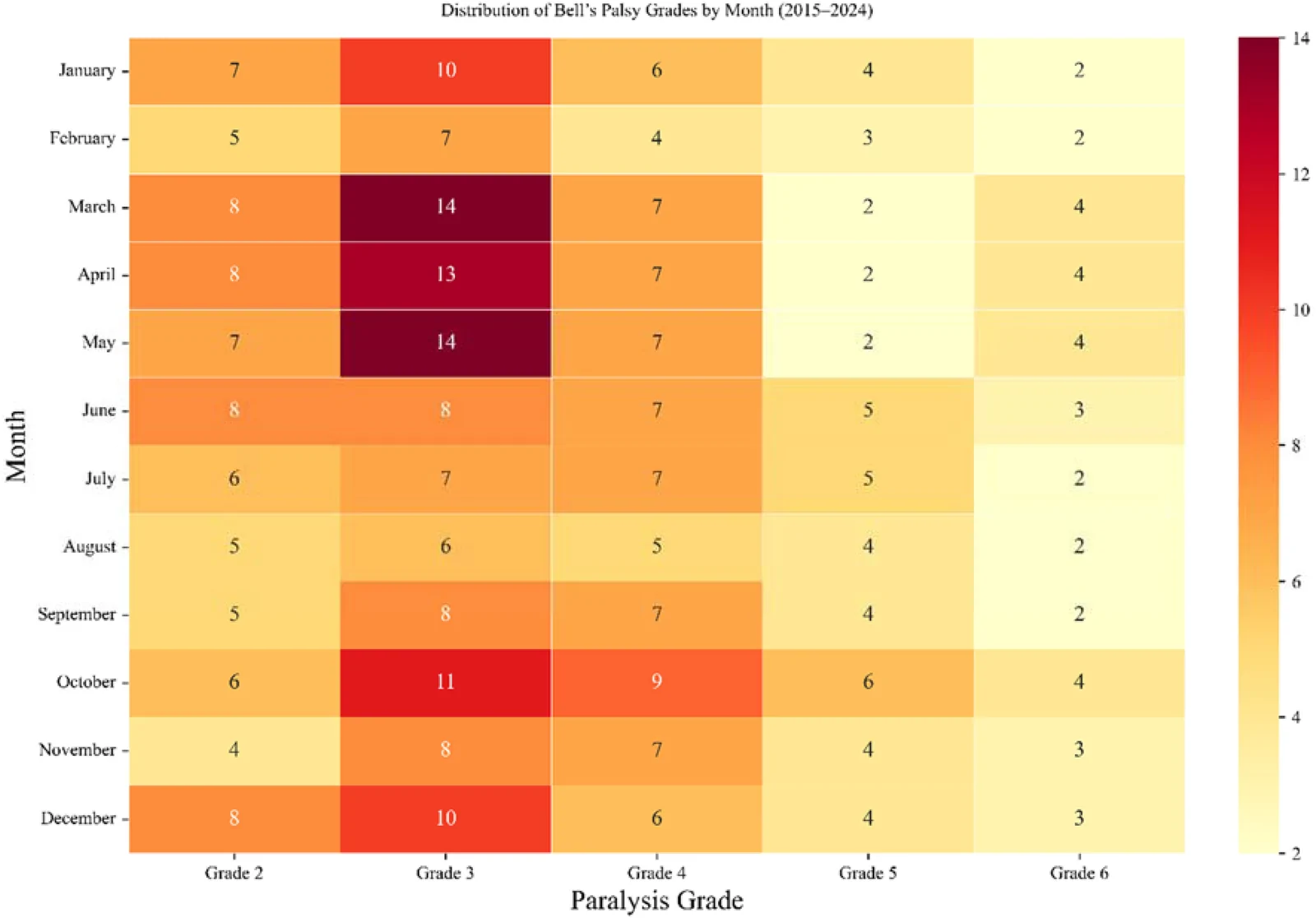
Heatmap illustrating the monthly distribution of Bell’s palsy severity grades.

**Fig. 4:**
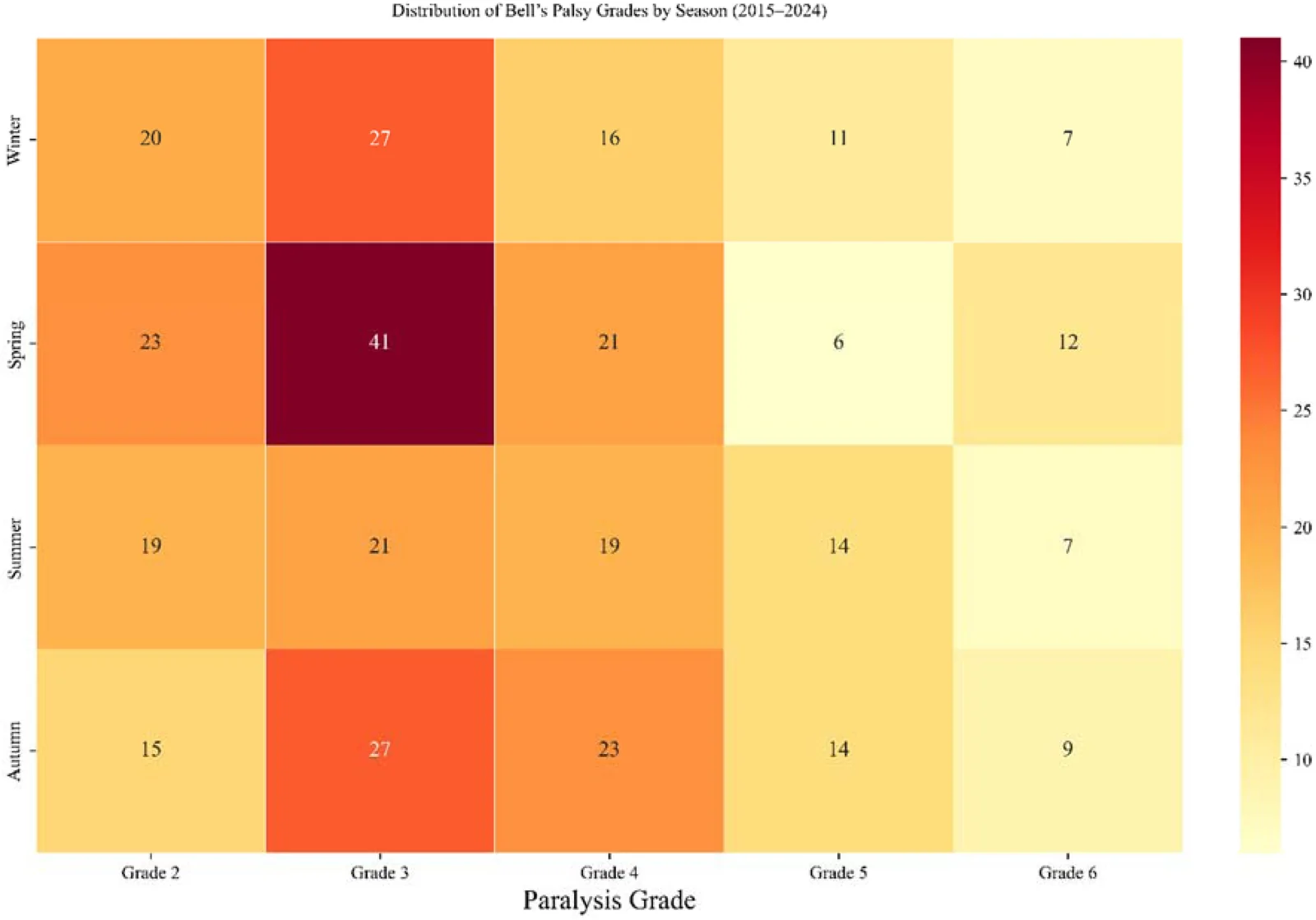
Heatmap illustrating the seasonal distribution of Bell’s palsy severity grades (2015–2024).

In the analysis of BP severity by month, the distribution of grades was similar across all months, and no statistically significant difference was observed (p = 0.292). This finding indicates that the severity of paralysis remained consistent among patients presenting during different months of the year (Fig. 3). Similarly, no statistically significant difference was observed in the distribution of paralysis severity by season (p = 0.471; Fig. 4).

## Discussion

In this retrospective study analyzing BP presentation dates over a 10-year period, data from 352 patients were evaluated. The mean age of the patients was 46.51-years, with a male predominance (53.98%). When analyzed by months, the highest number of presentations occurred in October, whereas the lowest was recorded in February. In the seasonal analysis, spring showed the highest number of cases, whereas the summer had the lowest. However, these differences were not statistically significant for either months or seasons. Similarly, no significant variation was observed among years. In the STL analysis, which enabled a more detailed evaluation using monthly case counts, small-amplitude, recurrent fluctuations were identified within the annual cycle of the seasonal component. These fluctuations were characterized by a modest increase that began in March compared with February and remained positive through April and May. However, the amplitude of seasonal variation was limited, and conventional monthly and seasonal comparisons showed no statistically significant differences. This finding suggests that although BP presentations may show minimal increases during certain periods of the year, there is no definitive evidence of a strong seasonal pattern. Therefore, the STL findings should be regarded as exploratory and hypothesis-generating rather than as confirmatory evidence of seasonality. The nonsignificant temporal trend observed in our study is consistent with several previous reports. However, the use of data from a single center limits the generalizability of our findings.^10^^,^^11^^,^^15^^,^^16^

Numerous studies have examined the relationship between BP and seasonal variations. Although many studies have reported a higher number of cases during colder periods, conflicting findings have also been documented.^10^^,^^11^^,^^15^^,^^16^^,^^18^ Magazi et al.^19^ reported that the incidence of BP was the highest during winter and the lowest during spring. Güneş and Karalı^20^ also reported a significant seasonal variation in case distribution, with the highest number of BP cases observed in winter and the lowest in summer. Similarly, Chen et al.^5^ reported that the lowest number of cases occurred in June, whereas the highest number occurred in December. Based on these findings, the authors suggested that exposure to low temperatures and high atmospheric pressure might act as risk factors for the development of BP. Consistent with these reports, Sonbay Yılmaz et al.,^6^ Hsieh et al.,^7^ Kar et al.,^8^ and Das et al.^9^ also reported the highest number of BP admissions during winter. In the study by Varga et al.,^10^ a higher incidence was observed during both winter and spring, which was interpreted as supporting evidence to the triggering effect of cold weather in BP pathogenesis. In contrast, Kim et al.^11^ conducted a study in South Korea and reported that BP admissions were more frequent during autumn. However, they also associated this pattern with low temperature and low relative humidity, emphasizing that transitional periods with sharp temperature drops, such as autumn, may be more influential than cold seasons themselves. Similarly, Ramsey et al.^12^ reported an increase in BP cases during autumn. Conversely, Köder et al.,^13^ in a study conducted in Türkiye, observed the highest number of BP cases during spring and summer, although the difference was not statistically significant. Salbaş et al.^14^ analyzed Google Trends data collected between 2004 and 2020 to investigate seasonal differences in online searches related to BP. They reported that search activity peaked during spring in the northern hemisphere and during winter in the southern hemisphere. The findings of our study are consistent with those reported by Köder et al.^13^ and Salbaş et al.^14^ The fact that different results have been reported even in studies conducted within the same country suggests that seasonal categories alone may not adequately explain the temporal distribution of BP. Collectively, these findings indicate that the seasonal distribution of BP is heterogeneous and may depend on population structure, climate characteristics, case definition, healthcare access, and the statistical methods used to evaluate temporal variation.^5^^,^^11^^,^^15^^,^^16^^,^^21^

Several recent studies have used meteorological or environmental datasets, including temperature, atmospheric pressure, humidity, rainfall, solar radiation, and air pollution indices, to investigate potential environmental associations with BP.^5^^,^^21^, ^22^, ^23^ Because such direct exposure variables were not available in the present dataset, our findings should not be interpreted as evidence supporting or refuting specific environmental triggers. Rather, the present results indicate that no clear monthly, seasonal, or annual clustering was identified among patients presenting to our tertiary otorhinolaryngology clinic.

Initial facial paralysis severity is clinically relevant because higher House-Brackmann grades have been associated with prognosis and recovery outcomes in BP.^24^, ^25^, ^26^ Therefore, evaluating severity according to month and season may provide information beyond simple case counts, even when no significant temporal differences are detected. In our study, the distribution of paralysis severity was also examined, and similarly, no significant differences were identified. To our knowledge, only two studies have specifically investigated the relationship between BP severity and seasonal variables. Among these studies, Hsieh et al.^7^ reported no significant association between paralysis severity and seasonal factors, consistent with our findings. In contrast, Güneş and Karalı found that higher-grade BP was more frequently observed during autumn and winter and that recovery rates among patients with advanced-stage BP were lower during these seasons.^20^ In our study, the absence of a temporal relationship with paralysis severity suggests that seasonal variables may not be a major determinant of disease severity, although individual factors such as comorbidities and time to presentation may also have influenced clinical severity. This observation further supports the concept that BP is a multifactorial disorder with a largely idiopathic nature.

The COVID-19 period may also have influenced healthcare-seeking behavior, outpatient accessibility, referral pathways, and the timing of presentation for acute facial paralysis. Additionally, peripheral facial palsy has been suggested to occur in association with both SARS-CoV-2 infection and COVID-19 vaccination, which may have further influenced case distribution during the pandemic period.^27^, ^28^, ^29^, ^30^, ^31^ Accordingly, annual fluctuations observed during or after the pandemic period should be interpreted cautiously, particularly in presentation-based retrospective studies.

The strengths of our study include the analysis of a 10-year period and the use of STL analysis, which allows a more detailed and comprehensive evaluation beyond conventional statistical methods. However, the study has certain limitations. First, this was a single-center, clinic-based study and did not include a predefined population denominator; therefore, the findings should not be interpreted as population-based incidence estimates. Second, direct meteorological and environmental exposure variables were not analyzed, limiting our ability to evaluate potential environmental triggers of BP. Third, although 352 patients were included overall, the number of cases became relatively limited after stratification by month, season, year, and House-Brackmann grade. This limitation may have reduced the statistical power to detect modest seasonal differences and may render minor recurrent fluctuations in the STL seasonal component uncertain. Finally, although 18 patients were excluded because they presented more than 1-week after symptom onset, we were unable to reliably analyze onset-to-presentation intervals and delayed-presentation exclusions according to year or season for all initially screened patients because symptom onset dates were not consistently available in excluded records. Therefore, a potential selection effect related to delayed healthcare-seeking behavior, referral patterns, healthcare access, and onset-to-presentation intervals, particularly during the COVID-19 period, cannot be completely excluded.

## Conclusions

This 10-year single-center retrospective study evaluated clinic-based temporal presentation patterns of BP using conventional categorical analyses and STL analysis. No statistically significant monthly, seasonal, or annual variation was identified, and paralysis severity did not differ according to temporal categories. STL findings suggested only small-amplitude recurrent fluctuations, with a modest increase that began in March compared with February and remained positive through April and May; however, these findings should be interpreted cautiously because of the clinic-based design, relatively limited subgroup counts, and the absence of direct meteorological or environmental exposure data. Future population-based, multicenter studies incorporating meteorological, air pollution, viral activity, and healthcare utilization data are needed to clarify whether environmental factors contribute to the onset or severity of BP.

## ORCID ID

Vural Akın: 0000-0002-0050-4837

Hasan Yasan: 0000-0002-5470-6784

Gökhan Keskin: 0009-0008-8760-0258

Furkan Yeşilkuş: 0009-0008-4871-0200

Mehmet Emre Sivrice: 0000-0002-2396-6794

Yusuf Çağdaş Kumbul: 0000-0002-0713-2933

## Declaration of Generative AI and AI-assisted technologies in the writing process

Artificial intelligence tools were utilized during the data analysis phase to verify Python code developed in Google Colab and to provide grammatical support during the English translation process. No study-related content, including study design or manuscript content, was generated using artificial intelligence tools. The manuscript was subsequently reviewed, edited, and certified for language accuracy by native English-speaking professionals.

## Funding

None.

## Data availability statement

The authors declare that all data are available in repository.

## Previous presentations

None.

## Declaration of competing interest

The authors declare no conflicts of interest.

## References

[1] Zhang W., Xu L., Luo T., Wu F., Zhao B., Li X. (2020). The etiology of Bell’s palsy: a review. J Neurol..

[2] Kim S.H., Kwak M.Y. (2025). Update on medical management of acute peripheral facial palsy. J Audiol Otol..

[3] Dalrymple S.N., Row J.H., Gazewood J. (2023). Bell palsy: rapid evidence review. Am Fam Physician..

[4] Gardner S., Garber L., Grossi J. (2025). Bell’s palsy: description, diagnosis, and current management. Cureus..

[5] Chen J., Yu Z., Zhou W. (2023). Effect of temperature and air pressure on the incidence of Bell’s palsy in Hangzhou: a distributed lag non-linear analysis. Sci Rep..

[6] Sonbay Yilmaz N.D., Gur O.E., Kucuktepe U., Ensari N., Yilmaz M.D. (2019). Seasonal distribution of the incidence of Bell’s palsy. Med Science..

[7] Hsieh R.L., Wang L.Y., Lee W.C. (2013). Correlation between the incidence and severity of Bell’s palsy and seasonal variations in Taiwan. Int J Neurosci..

[8] Kar M., Altıntaş M. (2022). Seasonal distribution of Bell’s palsy. Indian J Otolaryngol Head Neck Surg..

[9] Das P., Chowdhury N.H., Hasan A.A., Kalam Azad M.A., Ilias A. (2021). A Study on seasonal variation of Bell’s palsy in a district area of Bangladesh. Med Today..

[10] Varga E., Battamir U., Szegedi I., Hudák L., Kovács N., Nagy A.C. (2023). Seasonal patterns in the epidemiology of Bell’s palsy in Hungary. Front Neurol..

[11] Kim M.H., Park S.Y. (2021). Population-based study and a scoping review for the epidemiology and seasonality in and effect of weather on Bell’s palsy. Sci Rep..

[12] Ramsey D.J., Haas L.P., Tucker S.M. (2022). Long-term outcome after acute peripheral facial palsy. Ophthalmic Plast Reconstr Surg..

[13] Köder A., Belada A., Kılıçaslan S. (2025). Seasonal pattern of Bell’s Palsy incidence in a post-COVID-19 context. Duzce Med J..

[14] Salbaş E., Ketenci S. (2022). Infodemiology of Bell’s palsy: tracing the seasonality of facial paralysis. J Back Musculoskelet Rehabil..

[15] Aljarallah B.F., Alolayqi F.S., Alghizzi B.A. (2025). Seasonal patterns in Bell’s palsy: a systematic review and meta-analysis. Front Neurol..

[16] Jeong J., Chung J.H., Ryu S. (2024). Monthly variation in Bell’s palsy based on population data of Korea. Audiol Neurootol..

[17] Cleveland R.B., Cleveland W.S., McRae J.E., Terpenning I. (1990). STL: A seasonal-trend decomposition procedure based on loess. J Off Stat..

[18] Alanazi F., Kashoo F.Z., Alduhishy A., Aldaihan M., Ahmad F., Alanazi A. (2022). Incidence rate, risk factors, and management of Bell’s palsy in the Qurayyat region of Saudi Arabia. PeerJ..

[19] Magazi D., Longombenza B., Mda S. (2020). HIV infection, seasonality and younger age predicting incident Bell’s palsy among black South Africans. BMC Neurol..

[20] Güneş A., Karalı E. (2020). The effect of seasonal changes on frequency and stage in Bell’s palsy. Bozok Med J..

[21] Zhang L.Y., Jiang M.Z., Li D.M. (2024). Non-linear association between climatic parameters and Bell’s palsy prevalence of hospital outpatients: An ecological proof in Kunshan, Suzhou, China. Dose Response..

[22] Zhang C., Dong F., Wu Q. (2023). Sunshine duration and solar radiation contributed to severe Bell’s palsy: an 11-year time series analysis based on a distributed lag non-linear model model. Medicine (Baltimore)..

[23] Kim S.Y., Min C., Choi J., Park B., Choi H.G. (2020). Air pollution by NO2 is associated with the risk of Bell’s palsy: a nested case-controlled study. Sci Rep..

[24] Yoo M.C., Park D.C., Yeo S.G. (2021). Association between initial severity of facial weakness and outcomes of Bell’s palsy. J Clin Med..

[25] Kafle D.R., Thakur S.K. (2021). Evaluation of prognostic factors in patients with Bell’s palsy. Brain Behav..

[26] Goo B., Kim J.H., Park J., Baek Y.H., Nam S.S. (2025). Recovery rate and prognostic factors of peripheral facial palsy treated with integrative medicine treatment: a retrospective study. Front Neurol..

[27] Kwak M.Y., Lee H.Y., Lee S.A. (2024). The impact of the COVID-19 pandemic on Bell’s palsy and Ramsay-Hunt syndrome: a multicenter retrospective study. J Korean Med Sci..

[28] Tamaki A., Cabrera C.I., Li S. (2021). Incidence of Bell palsy in patients with COVID-19. JAMA Otolaryngol Head Neck Surg..

[29] Rafati A., Pasebani Y., Jameie M. (2023). Association of SARS-CoV-2 vaccination or infection with Bell palsy: a systematic review and meta-analysis. JAMA Otolaryngol Head Neck Surg..

[30] Lee S., Lee N.K., Lee S.W., Kim Y.J. (2025). Changes in the nationwide incidence of Bell’s palsy in the general population before and after the COVID-19 pandemic: An ecological study. J Med Virol..

[31] Afza T., Kumar V.K.C., Subramanian S.S. (2026). Bell palsy incidence in patients with post-COVID: a retrospective study. Int J Nutr Pharmacol Neurol Dis..

